# Historical perspectives in medical humanities: two views on vaccination during the plague pandemic in Calcutta

**DOI:** 10.1007/s40592-025-00251-4

**Published:** 2025-06-03

**Authors:** Utsa Bose

**Affiliations:** Faculty of History, University of Oxford, Oxford, UK

**Keywords:** Vaccine, Vaccine hesitancy, Pandemic, History, South Asia, Medical humanities, Bioethics

## Abstract

The aim of this article is to complicate and rethink histories of non-Western responses to vaccination, as well as to see how a historical perspective may contribute to contemporary discussions of vaccines within the medical humanities. This is done through a case study approach. The focus of this article is a collection of essays written by a Bengali prophet-astrologer and published during the plague pandemic in Calcutta, British India, at the turn of the twentieth century (1899). While one essay in the collection was critical of the plague vaccine, another essay, in a later section of the same collection, celebrated the vaccine and its developer Waldemar M. Haffkine. The first part of the article situates the context of the text’s production, as well as the background of the author. In the second part, it analyses the reasons *why* the plague vaccine was criticised. In the third part, it looks at how the author celebrated the plague vaccine in a later section of the collection. In the fourth section, it attempts to answer why the astrologer changed his view on vaccination. Finally, in the conclusion, it discusses how this case study intervenes historiographically, and how certain themes raised by the source persist today, reiterating the importance of a historical perspective.

## Introduction

Discussions on vaccination are rife within the field of medical humanities. Within these discussions, one of the main questions that often concerns scholars is: what are the reasons people may object to vaccination, how does one deal with it? It is here that my article intervenes. It asks two questions—Firstly, how can historical sources, rooted in non-Western contexts, help us understand objections to, and acceptance of vaccines? And secondly, how can such examples help us rethink not only the way vaccine histories are remembered and narrated, but also how we understand attitudes towards vaccines in the present? To answer these questions, I use a historical case study, wherein a native colonial subject both initially rejected and eventually accepted the same vaccine during a pandemic. The focus of this article is a collection of essays written by a Bengali astrologer, published in 1899, at the height of the plague pandemic in Calcutta, British India. In the first sections of this article, I situate and contextualise the author in question. In the second section, I look at the many reasons which may have contributed to his objection to the plague vaccine in Calcutta. In the third section, I look at how the same author accepted and even celebrated the same vaccine in a later section within the same collection of essays. Finally, I look at the reasons why the author in question may have changed their mind about vaccination. In the conclusion, I look at how this case study can help us rethink not only certain framings of colonial history but also help us in contemporary discussions on vaccination. Using this example, I make a case for the importance of a historical perspective in the medical humanities, I do so by tracing similarities between the ways in which a vaccine was viewed in this previous epidemic, and certain contemporary discussions on vaccination.

The past is never past. For policymakers and healthcare professionals alike, past precedence often dictates the choice of a particular intervention. Historical perspectives help in showing how certain interventions worked or did not work across time; in doing so, they challenge certain assumptions and biases about the past, laying bare continuities and discontinuities between circumstances, contexts, and periods. They may prove instructive in this way, and the failure of certain policies may often also be a result of a certain kind of historical amnesia, which fails to consider similar (failed) attempts in the past. There have thus been discussions of the need for a historical perspective, for “putting the past back in” in discussions of public health policy in the present (Stevens et al. 2006). Such a notion is built upon the assumption that the past continues within the present and can help us place the present within a larger trajectory. An example of the recognition of such a function of history is the World Health Organization (WHO)’s Global Health Histories (GHH) project. Founded in 2004, the “enduring rationale [of the GHH] has been that understanding the history of health helps the global public health community to respond to the challenges of today, thereby shaping a healthier future” (Bhattacharya et al. 2020). Similarly, a recent study has argued that pandemic preparedness should incorporate the humanities and the social sciences since they “open opportunities to deepen an understanding of how cultural and social values and traditions shaping lives and belief systems could affect and perhaps even enhance vaccine acceptance” (Frampton et al. 2024).

Yet, comparisons may also have their problems, and thinking only comparatively may engender its own set of issues. As it has been pointed out, the overemphasis on finding comparisons between the past and the present may prove problematic, since policymakers, academics and scientists may, often, moved by the “comfort of comparison”, force parallels between periods, and end up glossing over the novelty of a present moment (Lachenal and Thomas 2020). As Charles Rosenberg succinctly puts it, three of history’s core principles are “context, context, and context” (1999), and uncritical comparisons, in their quest to find similarities, betray the historiographical process by often decontextualising historical sources. Acts of comparisons do not mean that we merely use concerns raised in the past in a decontextualised way and connect them to present discussions. The use of historical precedents then, must be seen for their instructive utility, but one must also be cognizant of the limitations of comparisons. While my article does not directly use a historical example to make a case for public health policy, or speak of the future, it shares with these ideas discussed above the notion that an understanding of the past can help us navigate and nuance certain abiding public health concerns today, specifically in the context of vaccination. Finding commonalities between attitudes towards vaccination during the plague in Calcutta at the turn of the twentieth century with attitudes towards vaccination today does not mean, therefore, the collapsing or ironing out the historical context or the novelty of the present. Rather, in this article, I have tried to stay true to the historical context of the text’s production and consider some of the *underlying issues* that undergirded many of the reasons for vaccine objection and acceptance. It is in these set of underlying concerns that, I argue, we may find traces of older concerns which persist today.

The historical context of this article is the so-called third bubonic plague pandemic. The so-called third bubonic plague pandemic—believed to have originated in southern China—reached British Hong Kong in 1894, from where it travelled to Bombay, British India in 1896. From Bombay, it soon spread to other cities in British India. Among these cities, I focus on Calcutta, which was not only a major port, but also the capital of British India until 1911. Although the disease soon spread to other countries, it was British India which saw the largest number of casualties—by some estimates, almost 9 million people died of the plague in British India between 1896 and 1914 with the death and devastation in some provinces (mainly the north and the west) being disproportionately worse than others (Arnold 1993). At the same time, British India was also the scientific theatre of the epidemic, as it was here that the vaccine for the disease was mainly devised, developed and largely put to use (Chakrabarti 2012). Historiographically, this essay contributes to a growing body of medical history in South Asia which attempts to rethink native responses to vaccination. This is in part because reactions against and responses to vaccination in this context has been largely viewed as forms of “resistance” or “protest”, a “resistance” inflected by “cultural” or “religious” beliefs (Arnold 1993). Although the importance of studying alternate worldviews and their response to vaccine technologies cannot be denied, such framings, while stressing on the importance of the native colonial subject’s agency and beliefs, traps them in a different kind of damaging epistemic binary—that of non-Western religious and cultural belief against the juggernaut of Western “science” and “reason”. Such framings, then, generally overlook the fact that there were many anxieties regarding vaccination in this period which cannot be explained only through cultural and religious difference. This does not mean that these differences did not exist, but there were also other practical reasons, reasons which cannot be seen only as religious and/or cultural difference, which made native subjects refuse vaccination (Bhattacharya et al. 2005). To this effect, it also contributes to the literature which critically assesses and rethinks vaccine hesitancy in the present, by engaging with the rationales by which vaccines are refused/rejected. (Hobson-West 2017).

## Situating the astrologer: Tarini Prasad as an “Influencer”?

The focus of this article is a collection of essays titled “*Plague-Sanhitā ba Aryaswasthyabidhān*” (“The Plague-Sanhita or The Aryan Hygiene”) written by a certain Tarini Prasad Jyotishi. Published in 1899 and running over 150 pages, Tarini Prasad’s text contained prophecies, essays on astral influences, ways of protecting oneself from the disease, health guidelines, and social commentary (Jyotishi 1899). It thus straddled the worlds between the medicinal, the divine, the astral, the cultural and the sociopolitical.

Sections of Tarini Prasad’s text have also been studied by the historian Dipesh Chakrabarty. In his essay, Chakrabarty differentiates between two kinds of understandings of the body. The first, characterised by colonial modernity and backed by the state, is the “individual body”. This view sees epidemic disease as affecting individual bodies and thus intervenes by targeting the individual body through public health measures such as vaccination. On the other side of the spectrum, Chakrabarty places the “social body”. This understanding of the body places epidemic disease as blights on entire communities, where epidemic causation is not merely material or biomedical, but also social and/or divinely ordained. This latter view includes cults of disease goddesses in South Asia, where the disease is not seen as affecting individual bodies, but as affecting the body of the community (the “social body”) as a whole, and it is only through intervention at a community level—through acts of community propitiation, ritual, change in social habits, etc. that the epidemic can be stemmed. These two views of the body, to Chakrabarty, are often in contestation with each other, the latter seen as subaltern voices against the bulldozer of modernity. Here Chakrabarty takes Tarini’s view of the plague and its vaccine as an example of the “social body”, evidenced by a “religious mode of thinking” and “opposition to the English”. To this effect, Chakrabarty quotes sections from Tarini’s text (which he states was written by “an astrologer”) wherein the astrologer rejects the plague vaccine on the grounds that the susceptibility to the plague may also be due to moral failure and also rejects the injecting of the plague poison into the body on the grounds that the “British have not succeeded [as of yet] in discovering certain remedies for the plague or cholera”. This view also sees the coming of the plague as a result of the sin of colonial modernity (2012). The invocation of the social body, the rejection of the plague vaccine, and the astrologer’s critique of colonial “foreignness” is thus seen as an instance of subaltern resistance against the juggernaut-like influence of colonial modernity, as well as an example of an “anti-statist mood” (Chakrabarty 2012).^1^ This reading of the astrologer’s text, while interesting and illuminating, is incomplete for a few reasons. Firstly, merely suggesting that the text was written by “an astrologer” belies the importance this figure held not only in his own society, but, as I will show, in a larger Anglo-American context as well. This has implications on the question of influence, and the range of people who might have been reading the text. Secondly, reading the astrologer’s rejection of vaccination as a “religious mode of thinking" and as "opposition to the British” misses the many other ways in which his initial resistance to vaccination may be understood. Additionally, singling out his critique of vaccination (and not commenting on his eventual acceptance and celebration of vaccination) leads not only to a decontextualised reading of the text, but also reinforces certain binaries—subalternity vs. state power/radical ideas of difference. Finally, eliding the same figure’s acceptance of the vaccine misses a crucial point—that a historical actor may change their mind about a state-backed medical intervention, albeit for several reasons. This eliding, in my view, is probably due to the second point mentioned above, which looks at the astrologer as a “subaltern” figure and stresses on difference and contestation.

Tarini Prasad was a prominent figure in the realm of Hindu astrology, the news of whom spread far and wide, across the Anglo-American world, from Bombay, to Kansas and New York. He was often titled the “Bengali Zadkiel” or even the “Prophet of Bengal” (*Times of India* [hereafter *TOI*] 1893)—the original “Zadkiel” was a certain Englishman dubbed “the most notorious astrologer in Britain”, who published the Zadkiel’s Almanac in London in the middle of the nineteenth century (Anderson 2005). In 1897, it was said that Tarini had predicted the “Jubilee Earthquake”—dubbed so since the earthquake had coincided with the Diamond Jubilee of Queen Victoria in 1897—correctly. This popularised him further, and the TOI conducted an interview with the “doyen of Hindu astrologers” (1897). With this background in mind, it must be understood that by the time Tarini Prasad’s manual on the plague was published in 1899, he was already an influential, known figure in elite circles, making the intended audience quite large in number. In different points in the collection, the astrologer addressed his readers, and in one instance (mentioned later) he referred to one of his prophecies printed in an older newspaper (Jyotishi 1899) which suggests that the astrologer not only had a large audience in mind when he was writing his text, but also that he assumed that many of the readers of this text would have read his prophecies printed in newspapers. All of which suggest that his views on the plague (and plague vaccination) would have potentially been taken seriously.

## Doubt: questioning the plague vaccine

In his introduction to the text, the astrologer had mentioned a few prophecies about the “distant future” of the plague. One among these read

“the plague vaccine will be of no use against the disease” (Jyotishi 1899).

The story of vaccination—and specifically Haffkine’s vaccines—in Calcutta had a longer history. In 1894, Waldermar M. Haffkine, the Russian Jewish bacteriologist, had begun experimenting his cholera vaccine on slum-dwellers in the city, which, while successful to some extent, also had other risks such as side effects and could not provide complete protection from the disease. Thus, it would not be an oversight to suggest that his vaccine could have been seen by some as questionable. However, in 1896, Haffkine was commissioned by the Government of India to start work on the plague vaccine (Chakrabarti 2012). Calcutta, while being historically known for its epidemic outbreaks, was, since the early 1890s, caught in a great sociocultural and socioeconomic flux, characterised by urban overcrowding, an influx of migrant labourers, communal riots, and squalid health infrastructure. There was also a long-standing distrust between influential sections of the native population in the city and the colonial authorities on the question of urban planning and economic allocation, all of which complicated the reception of the state’s sanitary measures by the natives (Harrison 1994; Chakrabarty 1981). As the plague raged in Bombay, the Government of India, to curb the disease’s further spread, and, to send a signal to other countries, some of which were imposing damaging trade sanctions on India, created the Epidemic Diseases Act (EDA) in 1897. This Act allowed for the segregation of plague suspects, medical inspections, and house-to-house searches. It was widely criticised by the native population and led to violence, with Bombay being the worst affected by it (Harrison 1994). Fearing the disease’s spread into Calcutta, the authorities undertook vaccination drives in the city, and newspapers carried articles by researchers and plague authorities who cited statistical data to prove the vaccine’s high rate of efficacy (TOI 1898). In this background, when Calcutta was declared plague-stricken in May 1898, the declaration caused widespread consternation and a great exodus out of the city, as the natives feared the imposition of the same draconian segregation and house-to-house visitations that were imposed Bombay. Partly due to their negative experience in Bombay—the city had seen terrible violence against the plague measures—and due to the “mass exodus from the city”, segregation in Calcutta was almost abandoned (Harrison 1994). In its place, inoculation, it was argued by some, would be preferable to the people of the city (*Bengalee* 1898).^2^ However, there was, subsequently, a native fear that the government would “force” vaccination on its subjects, a fear that was compounded by a rumour that the pandemic, “imported from China”, and was meant to be a sacrifice for the “bloodthirsty gods”, which would be done by inoculating the natives through the “plague serum” (1899).^3^ In the report of J. Nield Cook, the Health Officer of Calcutta, it was specifically mentioned that the sacrifice would be to the goddess Kāli—an influential goddess in Bengal (1900).^4^ Before moving onto further details, it is interesting to note that the rationale which led to such an exodus and such anxiety and scepticism in Calcutta was based on, among other things, two issues. Firstly, it was based on the logic that the plague was a deadly disease—it had, after all, caused many deaths in Bombay—and that unjust plague policies, brought into effect elsewhere, would be transposed onto this city because a similar pandemic condition had been declared. Secondly, when vaccination, as an alternative was suggested, this led to a new set of fears. These new set of fears, inflected through religious and cultural imagery and imaginaries, were built on an underlying mistrust of government intentions. Thus, although the initial cause for alarm—the bringing into effect of the EDA in the same way in both places—was flagged, concerns soon morphed into something else—forceful vaccinations. This was primarily because the locus of concern was not only the disease and the draconian EDA measures, but also a distrust of the administration which was bringing into effect these plague protection measures. Thus, even after forceful evacuations and segregations were abandoned, vaccinations were seen with a degree of scepticism because it was the *same* administration which had brought into the segregation methods. There is much to be said about such acts of supplanting and substitution in the present, where concerns travel across interventions. Indeed, much of the panic over COVID-19, for instance, was based the evidence of examples of how the disease spread, about the evidence of its virulence elsewhere, and the nature of government measures elsewhere. At the same time, attitudes against vaccination were also often the result of a general loss of trust in the medical administration. In India, for instance, it has been suggested that the loss of trust in government, or healthcare institutions is one of the contributing factors to vaccine hesitancy (Paul et al. 2024).

Coming back to Calcutta, such alarmist narratives around the plague vaccine also fuelled riots in different localities, such as in the Muslim slums in the city (Harrison 1994). In response, the Government, together with the help of native gentry, began a campaign to allay the public. Many influential native men, along with their families and servants publicised their acts of getting vaccinated, which was meant to convince the population that the vaccine was not dangerous (Dhur 1898). The question of vaccination, and plague measures in general, however, also played into another set of concerns that were brimming in colonial Calcutta in this period—the conflict over issues of colonial modernity, and the anxiety over the range of influence the colonial state had on the private life of the native. These conflicts often spilled into disagreements not only between the native community and the colonial state, but also within different (elite) sections of the civil society, who disagreed on the form which “modernity” should take in the colony. In this context, the historian David Arnold has written how certain colonial laws such as the banning of *Sati* (ritual burning of the Hindu widow) or the banning of female infanticide was often interpreted by colonial native subjects as examples of the “arrogation of corporal power”, borne out of the state’s “latent claim” on the body of the native subject. The conquering of epidemics (through processes such as inoculation), too, was often interpreted as an extension of this claim (1988). The historian Partha Chatterjee has, similarly, written about a larger debate on the nature of modernity that was taking place between Bengali intellectuals at this time which spilled onto the questions of not only what kind of modernity would be worthwhile for the incumbent nation, but also of who should lead the people to that vision of modernity (2000).

It was within this milieu of commotion—anxieties over the epidemic, concerns over vaccination, and debates over the nature of (colonial) modernity itself—that Tarini published his collection of essays. Within this collection, in an essay titled “*Is the Plague Vaccine Warranted?*”, Tarini argued that “infecting a healthy body with the vaccine” was “not logical at all”, since “even with the vaccine”, “there were doubts regarding [its capacity to] reduce the spread of this infective disease”, and that, even after administration, “the poison [of plague] could not be stemmed”, since it “depended on differences in time, seasons, place, and personal failings/defects” (Jyotishi 1899). It is significant, then, that even though the government attempted to prove Haffkine’s vaccine’s efficacy by printing its high rates of efficacy in the newspaper, doubts over its effectiveness remained. In an earlier section, Tarini had argued that the plague had arrived in India since Indians had become immoral by adopting “foreign” (mainly colonial) customs and sociocultural habits, and consequently had forgotten who they were (Jyotishi 1899). The focus on morality was a common trope in astrological narratives, as the worlds of disease and sin were tied in astrological views of the world (Cunnigham and Grell 2000).

What are the many ways in which we can read Tarini’s critique of vaccination? On the first level, it was the questionable efficacy that made the astrologer wary of the vaccine. It is important to note that the astrologer was not giving credence to other rumours or popular narratives which suggested that the vaccine was poisonous; rather, he understood vaccines as being, essentially, poisons [*biṣa*], but poisons which were meant to kill other poisons. In an earlier section, Tarini had argued that

In this world…poisonous materials may also turn to become a force for human good; when administered correctly, even the *Halahāla* poison is beneficial to the human body. Again, Haffkine *saheb* [an honorific term for men in South Asia] opines that the plague-poison may be used to destroy the plague [disease]. Aryan physicians held an independent opinion [*svatantra mata*] called “poison-as-antidote” [*biṣam-biṣamauṣadhī*]… (Jyotishi 1899)

This reckoning with the ambivalence of poison/medicine may be traced back to ancient Indo-Chinese notions of relational toxicity, as well as, in the European context, on Paracelsus’s notion on poisons, where the toxicity depended correct dosage (Liu 2021; Arnold 2016; Gantenbein 2017). The example used to explain this poison-as-medicine, in Tarini’s case, was an ancient poison called *Halāhala*. The invocation of the ancient poison may be seen as an attempt at cultural authority, refracted by the astrologer’s experience as a native colonial subject and a devout Hindu. However, is this immediate subject position the only way we can read his critique of vaccination? He continued,

Just as poisoning a body to prevent it from being eaten by a wild animal is illogical, so too, the unnecessary injecting of a poison into a healthy body is illogical. The *Ṣāstras* [an ancient Hindu text] argue that it is only *after* symptoms/infections set that there is there a need to administer *Halāhala*. Then, what is the rationale behind injecting the poison into the body before symptoms set in, or doing so with the apprehension of infection? Even before coming up with definitive medicines for contagious diseases such as *cholera and plague*, you have started injecting their seeds into the human body… [italicization mine] (Jyotishi 1899).

Tarini, therefore, did not seem to have a problem with the administration of a poison, but rather with the *preventive* nature of the vaccine, which pre-infected the body before it had been attacked by the disease. Before analysing the implications of this critique of preventive medicine, it is important to notice that when Tarini criticised the technology of pre-infecting a body with the “seeds” of a disease, he mentioned cholera along with plague. As mentioned above, the cholera vaccine, too, had been developed by the same bacteriologist, Haffkine, and had been experimented upon in Tarini’s city, Calcutta, with limited results. What this criticism suggests, then, is that Tarini was perhaps also criticising a particular *group* of preventives—that of Haffkine’s vaccines. Thus, the mention of cholera along with plague suggests, in my reading, that the “you” in this particular case is not merely the “English” as a generalised whole—as Chakrabarty’s reading suggests—, but a particular “you”, Waldemar Haffkine. As I will show in a later part of this essay, this question of vaccine *source*—the question of *who* has developed the vaccine—is important, as when Tarini finally celebrated plague vaccine, he would also celebrate its developer, Haffkine.

Returning to his point about preventive medicine, how does one reckon with this critique of pre-infection? One way of reading Tarini Prasad’s wariness of vaccines may be to read it as a struggle against what the historian Bernice Hausman has called the state of being “pre-ill”, which vaccination makes possible. Vaccination, by making us “pre-ill” works on “medicalizing…our existence in an attempt to forestall illness”, which translates as a sort of confusion, as we do not know whether it makes us “healthy or sick” (2019). Continuing his critique of vaccination, Tarini went on to argue,

The immunity necessary to protect the human body exists in the human body itself…Growth and decay are maintained in a terse natural equilibrium within the human body. Life, death, health and illness are all essential parts of the human condition. What, then, is the logic of introducing something else into the human body?... (Jyotishi 1899).

This was a point about the importance of natural immunity, and also the importance of disease as an essential component of what it meant to be human. In this vision, the vaccine was the unnatural intruder, an intervening, interrupting foreigner in the world where life, death, disease and illness were held in a natural equilibrium. Indeed, one of the assumptions of modernity, which vaccination attempts to bring to life, is the belief in disease as a “thing of the past” and of “the future of human life without infectious disease” (Hausman 2019). By stressing on disease being a part of the human life, Tarini was rallying against, what seemed to him, an illogical dream. At the same time, by stressing on the body’s innate strength, the astrologer was also perhaps, along with rallying against certain assumptions of modernity, laying claim to a sense of “*Svadeśī ātmaśakti*” (indigenous innate strength), a religious-cultural idea employed by Bengalis in this period as an anticolonial rhetoric, as well as to bolster a sense of community strength (Mukharji 2009). Added to this, he was also laying claim rather common critique of vaccination, which saw the act of injection as unnatural, and a form of violation of nature. Stretching this critique across space, we find similar (but not congruent) critiques of vaccine-as-unnatural forwarded by vaccine protestors in Britain during the 1870s, where they argued that vaccination tampered with the natural development of the (divinely created) human body (Durbach 2005).

And yet, the astrologer did not stop here. He also countered the modern dream of a world free of diseases and of pre-infection by arguing that if apprehension or the possibility of contracting a disease was the basis on which one administered a vaccine—and indeed that is what it seemed to be—then, he continued sarcastically, what stopped one from pre-infecting a healthy body from *every* possible disease? This was, importantly, a point about the (logical) limits of vaccination. The questions here seemed to be “Where does vaccination stop? How many vaccines are enough vaccines?” This critique, while rallying against modernity, was also intimately tied to the astrologer’s understanding of the body in Hinduism, which, not only included disease as a necessary part of its ontology but imagined the body as a “*vyādhi-mandir*”, a “temple of diseases”, containing or holding the possibility of contracting any disease (Jyotishi 1899). If the body was a temple of every possible disease, then would one have to vaccinate oneself against every possible disease? Tarini buttressed this point by suggesting, sarcastically, that given this understanding of the body, it did not, then, seem illogical to “fortify this temple [of the body] with vaccines for 360 million [possible] diseases” (Jyotishi 1899). This is, of course, a hyperbole, but the underlying point here seems to be, as mentioned above, a point about the (logical) limits of vaccination. However, to reiterate, it is significant that a critique of the dream of a world free of diseases—a dream of modernity itself—is refracted through a particular understanding of the body—the Hindu body, a “temple of diseases”. Tarini then stated that he preferred not to question the opinions of those who had already taken the vaccine, and that his comments should be mulled over by those who were still unvaccinated, or on the fence about vaccination. Finally, he assured the readers that although the medicine against the infection had not been found yet, it was only logical that “if a disease arrives, so it will its medicine”, and that it was only a matter of time before the medicine arrives. (Jyotishi 1899).

It is tempting to read Tarini Prasad’s critique of vaccination as an example of an alternate locus of modernity not essentially rooted in “Western science”, argued by a non-western colonial subject. These are important frames, but merely packaging this critique as an example of historical difference, of a “religious way of thinking” or otherness makes us fall into the dangerous binary—western medical policy vs. “indigenous” resistance—mentioned at the beginning of this article. Thus, while it is important to historicise and note the context from which Tarini Prasad was writing, while it is important to note the cultural, religious and political context which birthed this text, it is also equally important to trace similarities that such arguments may have had with critiques of vaccination in other parts of the world in the same period. It is also important to see these critiques as not only critiques of coloniality and colonial modernity, but critiques against some of the general assumptions of modernity itself, given by a colonial subject. Doing so frees the text from the some of the limits imposed on it by focussing only on notions of subalternity, radical difference and alternate modernity. What’s more, as I chalk out in the conclusion, doing so makes possible certain nuanced comparisons between questions raised in the past and certain enduring concerns in the present.

## Acceptance? Celebrating the plague vaccine

Having noted his doubts about the vaccine, barely 25 pages later, in the same collection, Tarini Prasad went on to launch a passionate and bold defence of the plague vaccine and its creator, Waldemar Haffkine. *When* had the astrologer’s view on vaccination changed? Although *Plague-Sanhitā* was published in 1899—according to the National Archives of India records—it is difficult to know when each individual essay in the collection was written. In Tarini’s introductory prophecies—where he prophesised that the vaccine would be of no use—, he had mentioned an older prophecy about the “coming year”, about the plague reaching Bengal through the Southern Coasts and not the railway tracks. This same prophecy—foretelling the coming of the plague to Bengal “the next year”—seemed to have been published in a *TOI* report the previous year (1898). In his manual, referring to the 1898 prophecy, he said that “as we see, that [older] prophecy has come true today” (Jyotishi 1899).

Therefore, the “today” when he was writing the introduction in his collection was clearly 1899, and even in 1899, he believed that the vaccine would not work. However, it may be that the section in defence of the vaccine was written *after* he wrote his introduction, which was then added to the text, and the book was published thereafter. Thus, in an essay titled “*Plague Treatment, Differences in Medical Opinion and How to Protect Oneself from a Plague Attack*”, the astrologer argued that the plague had created a crisis of medical authority and a consequent scramble for “correct medication” within Calcutta. However, no medical system [allopathy, homeopathy and *Āyurveda* (Hindu traditional medicine) or *Hākimi* (Islamic) medicine] had been able to come up with correct medicines for the plague; at the same time, many of the doctors who had been brought in the deal with the disease were not interested in medication; some were merely studying the disease, while another group, in his view, were more like log-keepers, interested in recording plague deaths. There was also, finally, the fear of the vaccinators and medical examinations which added to the chaos in the city. “Everyone is slowly getting vaccinated”, he wrote. He then reflected, “perhaps the disease is truly kept at bay by vaccination. Everyone is of this opinion, and so, even in the absence of a doctor, the one and only Haffkine *saheb* [an male honorific in South Asia] has become the object of our gratefulness…In this terribly trying time, within this deluge of different medical opinions, we have taken him to be the man chosen by fate [*adṛṣṭabān puruṣa*]. Today, he leads the future-treatment [*bhabiṣyat-cikitsā*] of plague.” Tarini continued:

He [Haffkine] is both the future-doctor [*bhabiṣyat-cikitsaka*] and the prophet [*bhabisyat-baktā*] of the plague! Even when the future of the world seems dark, belief gives us strength; therefore, the people of the world, and especially fate-believing and calculative Indians owe a great debt to Haffkine. Whatever will happen in the future, will happen; but how many can work towards treatments which promise peace [*śāntilābha*]^5^ in the present? The clever person who can, studying space and time, speak of the future and develop treatments for the future—such a person is a truly marvellous for *Bhārata* [an ancient name of India]. (Jyotishi 1899)

Tarini then went on to corroborate his support of Haffkine’s prophetic status by saying that Haffkine was a “great Russian chemist” and that “Russians are fatalistic people and work on predictions and prophecies”. He concluded that Haffkine’s vaccine had sown the “seed of future wellbeing” [*bhabisyatmaṁgalera bīja*] in the country, which made him garner the praise of the British government, finally stating that

Those who get inoculated in time and in good health will, undoubtedly be protected from the plague and the inoculators. At present, the fear of inoculators is greater than that the fear of the disease itself. Therefore, according to the Government, if one was to inoculate themselves with Haffkine’s mantra-medicine-vaccine [*mantrauṣadha-tīkā*] then there will no cause for fear, and this has been said by the Government as a form of reassurance. People may get vaccinated by choice. (Jyotishi 1899).

The mention of the animosity towards and fear of plague inoculators here is significant, primarily because this could suggest that vaccinating, to Tarini Prasad, also meant freedom from the fear of these figures. As mentioned earlier, in Calcutta, a Government policy was soon introduced wherein vaccination would not be forced upon anyone and would instead be a voluntary decision. This was a form of reassurance, and Tarini restated this point. However, this question of choice was, to some extent, illusory, as the alternative to vaccination was segregation within plague camps or hospitalisation, which was largely feared. As mentioned earlier, it was believed that many would choose vaccination over the alternatives (Harrison 1994). At the same time, as mentioned earlier, one of the fears during this period was also the perceived harassment of the plague-inoculators, who were seen as troublesome figures who would go from house to house scaring and even foisting the vaccine upon people. Thus, vaccination would not be forced upon anyone, but if one was to get vaccinated, there would no cause for fear. Tarini’s mention of the greater fear of plague inoculators is important, as it suggests, then, that vaccination also served a social purpose—it immunised the vaccinated not only from (potentially) the disease and the fear of the disease, but also from the harassment of the inoculator themselves. Thus, the choice of inoculation could have been seen as a better than its alternative—segregation and harassment from inoculators—which seem to induce much greater fear, greater, as Tarini said, than the disease itself.

What is also interesting here is the astrologer’s translation and appropriation of the method and logic of vaccination into the language of prophecy. His criticism of vaccination had been born, in part, by its anticipatory nature, and, perhaps even because the plague vaccine had been developed by Haffkine, who had developed a questionable cholera vaccine in the past. However, in his passionate acceptance of vaccination, it was this very anticipatory element that Tarini took to be proof of Haffkine’s greatness. In a moment of pandemic panic, the astrologer projected vaccine research as important in the present *precisely because* of its “future-oriented” nature, which made the vaccine researcher a “prophet” [*bhaviṣyat-baktā*] (literally “the one who speaks of the future”). He could, perhaps, in Tarini’s view, both speak for the future as a prophet [*bhavisyat-bakta*] and affect the future as a future-doctor [*bhabisyat cikitsaka*]. His vaccine was sowing the seed of “future wellbeing” [*bhabisyatmaṁgal*], in the present. Projit Mukharji has argued that several indigenous doctors in Bengal, during the plague panic, wrote about the effect that faith had on the diseased body, allowing it to fight infection innately (Mukharji 2009). However, Tarini Prasad was *not* a doctor, and his argument seemed to revolve around the idea of vaccine and vaccine research *as* belief itself. In other words, while some medical practitioners argued for the importance of faith during the epidemic, they did not conflate it with medical technologies such as vaccination. In contrast, the novelty of Tarini Prasad’s argument lies in the fact that he saw vaccination and vaccine research as acts which engendered faith, and thus it was not the coexistence of faith and vaccine as mutually reinforcing but autochthonous entities, but vaccines as objects generating faith. Thus, although Tarini Prasad accepted and promoted vaccination, he did so by presenting the mechanism in his own terms—he turned Haffkine into a prophet and conceptually translated the vaccine [*tīkā*] into a mantra-medicine [*mantrauṣadha*]. Vaccines, and Haffkine’s words and work, were translated as foretelling eventual recovery, which gave people—especially fate-believing Indians—something to believe in, in the chaos and anxiety of the present. In other words, although the future was fated, it was precisely the proleptic and future-oriented nature of vaccine research during a pandemic^6^ that made it worthy of great commendation, because it was giving faith in the present, during a present that was otherwise so fraught, fractured and riddled with confusion. It was a medicine that was also a “mantra”. It is important here to reiterate the point about vaccine acceptance and the question of source. When Tarini finally accepted vaccination, his acceptance was also a celebration of *personality*; in other words, he did not merely defend the prophetic nature of vaccination, he also celebrated the prophetic nature of the vaccine developer.

## Vacillating on the vaccine question, or, why change your mind?

Why did the astrologer alter his view on vaccination, and why is such a question relevant today? Tarini himself noted how “everyone” was of the opinion that the disease was kept at bay by vaccination, and everyone seemed be “slowly getting vaccinated”. It is probable that Tarini reflected changing public attitudes towards vaccination, or perhaps he himself had been vaccinated or seen others vaccinated, and, subsequently, protected from the disease. It was also, as he mentioned, a way of immunising oneself perhaps not only from the disease, but from the fear of the plague inoculators.

However, interestingly, a survey of news reports about Tarini Prasad Jyotishi, published in the same year as the collection of essays, reveals a different channel of potential influence. Between January and June of 1899, news of Tarini Prasad’s prophecies was published in several newspapers across India and in the United Kingdom. Interestingly, many of these articles reported how Tarini’s prophecies now chiefly lauded Lord George Nathaniel Curzon, the incumbent Viceroy of India, who was due to arrive that year. On the 7th of January, the *TOI* reported that the astrologer had prophesied that the new Viceroy would “mix well with the people” and that he would “adopt a peaceful policy with regard to famine and plague”, while “curtailing unpopular measures and expenditure”(1899). Another such article was published in the *Bristol Mercury* a few weeks later (1899). The connection with Lord Curzon is further cemented by the fact that a few months later, on the Viceroy’s arrival to India, the *Derby Beacon*—Lord Curzon was also, interestingly, a native of Derbyshire—published an article on the astrologer, calling him the “Indian Zadkiel”. It went on to mention all the predictions that the astrologer had made regarding the Lady and Lord Curzon, ending the article by stating that

The Indian Zadkiel, we are further told, has sent a copy of his forecast of 1899 to the Viceroy, and has received the courteous thanks of Lord Curzon, who at the same time intimated the deep interest with which he had perused the predictions. (1899)

This information about an alleged correspondence between the astrologer and the Viceroy adds a potentially new political dimension to the question of influence and the text’s intended audience. Given that the text was published in 1899, the year he began his support for Lord Curzon, it is possible that although in early 1899 Tarini was critical of Haffkine’s vaccines, his defence of vaccination, and his glorification of Haffkine, was a form of possible political support, written after his liaison with the Viceroy, or as an act leading up to it. It is interesting to note that in the pages succeeding his critique of vaccination, Tarini does support the government’s anti-plague measures, even going so far write long passages lauding the European nurses who were engaged in plague duty (Jyotishi 1899).

Over the next decade, Tarini Prasad’s influence seemed to grow further, and the news regarding his prophecies travelled throughout Britain and the United States. The same article, printed in *Derby Beacon*, had a section of it reprinted in the *Kansas City Journal,* a month later. This time, it contained a sketch of Tarini, titled “*An Astrologer of Might*” (1899). While his prophecies continued to be published in the coming years, a biography of the astrologer was published in Calcutta in 1902, which was recorded in the *Catalogue of Books on the Occult Sciences*, in London (Leigh Gardner 1902). This record was also reproduced in the *New York Tribune* the year after (1903). Over the next decade, Tarini Prasad published a few monographs, all of them in support of the Crown, chief among them a “melody” on the Delhi Coronation Durbar in 1911, which had been held to commemorate the coronation of King George V (Jyotishi 1911). As this list of publications show, our astrologer grew to be an important figure in the years following his support of vaccination and his support of the Viceroy. How much of this rise in influence was a direct consequence of his changed stance on vaccination is, of course, a question that can never be fully answered. Yet it is significant that Tarini ends up being a supporter of the Government in the first decade of the twentieth century, and it is also significant that one of the first moments which capture this drift towards power is his support of the Government’s vaccination policies and research. Tarini Prasad, as argued earlier, was an influential figure, and his support of the plague vaccine must have, in no small measure, been helpful to the Government which was trying, in the initial years of the plague, to promote vaccination. While the use of “celebrity endorsers” to promote vaccination is also a method heavily used by Governments and international health bodies even today (Gavi 2025), evidence suggests that the question of “influence” might also turn the other way, when celebrities publicly critiquing a certain vaccine may adversely affect uptake rates (CNN 2023). While the connection between political beliefs and consequent vaccination uptake rates and vaccine policies has been documented (Wróblewski and Meler 2024), the possible correlation between changed views on vaccination and its connection to politics has also been documented in the present. A Reuters report from 2021, for instance, focussed on the career trajectory of Michael Yeadon, who was a “scientific researcher and vice president at drugs giant Pfizer Inc.”; he also “founded a successful biotech”, but “then his career took an unexpected turn”, wherein he became a prominent vaccine sceptic during the COVID-19 pandemic. While the report stated that “why Yeadon transformed” his views remained a “mystery”, his vaccine sceptic views appeared on websites linked with prominent political figures who were also vaccine sceptics (Reuters 2021).

The link between political belief and vaccine endorsement is, thus, is a much-discussed theme in the present. Getting back to our discussion of the astrologer, does this mean, then, that Tarini Prasad became a “celebrity endorser” of the colonial state, and was thus co-opted by the state? It is tempting to suggest that this change in stance was because the astrologer, had, between the first and the second essay, been co-opted by the colonial state, that his alternate modernity was bulldozed by a singular version of (state-backed) modernity, and his views altered by this co-option. It is not altogether untrue that Tarini does become, in a sense, a mouthpiece of the Government and supports the state in the years following the publication of his collection. However, what is significant is that this may have been for *several* reasons—including the possibility that he had seen the vaccine work on various people, because he had seen many people getting vaccinated, or even because felt that the need for reducing fears about the vaccine was important, since the fear of vaccines was trumping the fear of the disease itself, and because vaccination was better than its alternative—fear and harassment of the inoculators. What is also significant is the way in which the astrologer promoted vaccination. As far as Tarini was concerned, the vaccine developer (and the resultant vaccine) were objects of trust precisely because they were *not different* from him. Thus, instead of purely “secularising” his discourse, as far as he was concerned, it was the vaccine scientist who had to be changed into a prophet. In this move, the astrologer, while becoming, in some senses, an instrument of the state, had also turned the vaccine into a ritual object, severely complicating the assumption of unidirectional co-option. Finally, we can never underestimate the power of possible political support and greater influence as an incentive for changing one’s mind.

It is important to note here, too that perhaps most interestingly, all of the astrologer’s initial reasons for vaccine rejection are not exactly overturned in his eventual acceptance of the vaccine. Thus, the changing of mind is not without its caveats. At one level, the practical effect of this change of mind was a call for a shift from scepticism towards the vaccine to an acceptance of the vaccine. Yet, nowhere in his acceptance of the plague vaccine does the astrologer suggest that the initial cultural, philosophical and religious reasons for vaccine scepticism are not valid anymore. Instead, it seems more than likely that his initial cultural, religious and philosophical reasons for rejecting the plague vaccine might have existed alongside his eventual practical, political(?) and ritual celebration of vaccine. Thus, the neat binaries of anti/pro-vax do not fit onto Tarini Prasad at all, and what we have is a far more complicated, layered negotiation of choice, which warrants our attention for its very nuance and complexity.

## Lessons from history?

Tarini’s Prasad’s two views on vaccination shed light on the potential ways in which a particular vaccine was, historically, initially rejected and eventually celebrated at the height on a pandemic. While the aim of this essay is not to interrogate the *exact* reasons for this shift in opinion—such an act, may not, perhaps be possible, but neither is it, I believe, wholly necessary—the documenting of this shift is, firstly, more important. For by focussing on it, I attempt to make a point about the use of the historical record in discussions of health, medicine, and disease and in the medical humanities in general—the point about people *changing* their mind about a healthcare intervention (in this case, vaccination). And secondly, what is important is perhaps not so much exactly *which* reason contributed to the shift in opinion, but the very plural nature of this answer that is also the point—the fact that the astrologer’s initial criticism of the plague vaccine and his eventual celebration of it may have been due a *combination* of reasons, some of which were rooted to his particular place in time, his culture, religion and context, while others had parallels in other contexts as well. Thirdly, the fact that some of the astrologer’s reasons for vaccine rejection more than likely existed alongside his call for vaccine acceptance also disturbs the otherwise neat binaries of anti/pro vax.

From a historiographical perspective, this article argues for the need to rethink certain resistance vs. imposition binaries in histories of colonial medicine and science, as well as the need to resist the often-uncritical deployment of notions of “radical difference” or “subaltern science”. Instead, a closer look at certain figures and sources often reveal that objection to a form of state-backed medical intervention may not only have been an instance of subaltern resistance or to only preserve a notion of radical difference; rather, it may have been due to a combination of reasons, including largely practical ones –such as doubts over efficacy, the importance of natural immunity, etc. At the same time, vaccine acceptance may also not necessarily have been a result of unidirectional co-option by the colonial state. It may have been due to other reasons including but not limited to the evidence of seeing more people getting vaccinated, or of vaccination being a comparatively less fearful alternative. The mechanism of acceptance may also not have always been “scientific” either; indeed, Tarini’s example shows how the astrologer celebrated vaccine research as an example of divination, and projected the vaccine scientist as a prophet himself. At the same time, a change in perspective may also have taken place due to political reasons, which may not necessarily have had to only do with whether the “science” was working better or not, but rather *who* was doing the science, and what the potential advantages were of siding with those promoting vaccination. Finally, shifting the focus away from questions of radical difference, one also notices commonalities not only in between this example and certain critiques of vaccination given in this same period but in different political and cultural contexts, but one also finds echoes of them in contemporary discussions on vaccination.

From a modern bioethical perspective, then, this article argues for the importance of historical perspectives in the medical humanities and does by highlighting certain issues raised by the historical source which largely persist in contemporary discussions on vaccination. Thus, issues such as anxieties over questionable efficacy, doubts over a current vaccine which are based one’s previous experience from the same vaccine source, the abiding importance given to the role of disease, the fear of too many vaccines, or even the importance of natural immunity are still drivers against vaccine uptake today. Doubts of vaccine ineffectiveness largely persist; indeed, while one recent study on the COVID-19 vaccine in India suggested that the “lack in trust in vaccine protection” was a minor factor which contributed to the vaccine hesitancy and even refusal (Umakanthan et al. 2021), another study, undertaken during in 2021 during the COVID-19 pandemic, suggested that the lack of trust in vaccine efficacy as a contributing factor to the “stiff resistance” against mass vaccination of Indians even with the prospect of a third wave looming (Das and Mohanty 2021).^7^

It is often assumed that vaccine hesitancy or even refusal in the present is a result of ignorance about the realities of vaccine research and the vaccine’s efficacy; however, as has been increasingly acknowledged, vaccine objection may also be a result of a loss of trust in the medical experts themselves (Navin 2015). Thus, it has also been suggested, as argued earlier, that vaccine hesitancy, or even refusal, is often a result of a larger problem of eroding trust in healthcare institutions. The historian Elena Conis has noted that one of the ways in which the anti-vaccine movement in the USA took shape was as a movement against “Big Pharma”; Conis has rightly noted that this fight against “Big Pharma” was part of a “larger backlash against transnational corporations”, a critique which was part of the backlash against globalisation, where these corporations were also seen as damaging the environment and disregarding consumer safety in their profit-driven goals. At the same time, older drugs often developed by these same corporations were also increasingly found, in some instances, to cause certain medical complications, all of which added to resistance against vaccines developed by them (2014). Although Tarini’s critique of the plague vaccine was not, of course, against such large corporations, it is significant that one of the probable causes he was vary of it seemed to be possibly due to older experiences, because the plague vaccine had been developed by the same man who had also developed a questionable cholera vaccine in the recent past.

At the same time, as Hausman argues, the critiques of vaccination as unnatural, or of disease being a constituent part of human life, are abiding concerns which animate anti-vaccination sentiments even today—Hausman uses the United States as her case study—, as many who oppose vaccination do so because they believe in the “transformative” and “valuable” role of disease in “the calculus of human immunity, survival and thriving” (2019). It has also been argued that one of the motivations behind anti-vaccination sentiments may be a commitment towards natural parenting (Navin 2015), which thus sees vaccines as unnatural intruders in an otherwise natural order. Finally, many parents hesitate to/resist vaccination for their children because of the perceived problem of vaccination drives today being seen as “too many, too soon” (Rodriguez 2016); the argument here is against the perceived high number and frequency of childhood vaccination, which is seen as problematic, a fear compounded by anxieties regarding too many vaccinations potentially reducing or overwhelming the infants natural immune system, and although studies have questioned and tried to disprove these fears, they still persist (Gellin et al. 2000; Offit et al 2002). Although Tarini does not speak of the “too many, too soon” in exactly the same way, there is an underlying problem of limits—the questions “Where does vaccination stop? How many vaccines are enough vaccines?”— which carries through.

There are also certain parallels in the ways in which the astrologer eventually accepted vaccination too. Today, as in the past, vaccination uptake may increase not particularly due to an increase in one’s trust in science or even the state, but because vaccination may be seen as a better alternative to a worse inconvenience which might accrue if one is not vaccinated. To promote mass vaccination, some suggest a certain degree of coercion whereby although vaccination is not made mandatory, those unvaccinated are “permissibly excluded from public services, certain forms of employment, or pay fines for failure to comply with vaccine requirements” (Flanigan 2014). Often, the practical upshot of such decisions, if taken, results in an uptake of vaccination, since vaccination is seen as better than the price one must pay for not vaccinating. At the same time, an increase in “hope” has increasingly been seen as a positive factor which reduces vaccine hesitancy and increases vaccine uptake (Adam et al. 2022). To this effect, a recent exhibition titled “*Injecting Hope: The Race for a COVID-19 Vaccine*” showcased the challenges and triumphs while developing the COVID-19 vaccine (Pandemic Sciences Institute 2024). The focus on hope has parallels with Tarini Prasad’s point about the importance of vaccine research as a form of hope in an anxious pandemic present. At the same time, representations of vaccines as faith-ritual objects are also key drivers for increasing vaccine uptake in some contexts. A case study from a COVID-19 vaccine drive in Bhutan shows how the government used Buddhist astrologers to perform divination and rituals to positively affect COVID vaccine uptake. Thus, vaccine drives often took place on certain ritually imbued days (according to astrologer calendars), while certain mantras and ritual medicines were distributed along with vaccinations (Tashi 2022). Finally, entanglements between political affiliations and vaccine scepticism are also important concerns in contemporary discussions of vaccination.

As my article shows, several abiding, contemporary concerns about vaccination were also present in the past, and therefore, a historical perspective may prove useful in contemporary discussions of vaccinations. Does this mean that we take “lessons” from history? While I hesitate to consign to history such as straightforward didactic role, it is interesting and important to note that although we have moved far from Tarini’s point in time, and his cultural context is also situated and specific, what persists in the form of echoes between this historical example and present discussions on vaccination are repetitive patterns of behaviour, certain choices and certain anxieties—repetitive in the sense that they are prompted by the similar underlying fears, concerns, worries and motivations. By highlighting the endurance of such concerns, historical perspective may help us place many otherwise seemingly immediate and contemporary concerns within a larger canvas of human losses and victories, of successes and sufferings. However, having drawn certain similarities, one must, as mentioned earlier, be wary of the “comfort of comparison” for there is much in the present that does not, and indeed cannot neatly fit onto patterns in the past, and neither is it the historian’s job to merely reduce historical examples to echoes or comparative frames. The present, too, is novel in its own right, and cannot solely rely on guidance on past behaviour, although such examples may prove, to an extent, instructive. The past is muddy, nuanced, and seemingly contradictory. If one of the aims of historical analyses is to find parallels, one of its other aims is laying bare this muddiness. It is the act of suggesting that in many cases, the reasons for certain choices, the reason for certain alliances and rejections are unclear, unclear not in that one does not have an answer, but in that there is often no *one, clear answer.* A person may hold vaccine hesitant sentiments, even rejecting a certain vaccine, and then the same person may then go onto accept the same vaccine, but the reasons for this initial rejection and eventual acceptance views are almost always more layered and complex than labels of anti/pro-vax allow. To bring historical perspectives into the medical humanities, thus, it seems to me, means recognizing and reconciling with the cohabitation of similarities and incongruence between the past and the present. Yet, incongruence, like similarities, may also be generative. The present, too, is layered, muddy, and complicated. While the recognition of historical examples can help us nuance our present discussions, recognising the muddiness of the past can prove to be, also, an instrument of empathy, making the anxieties, and even muddiness of the present seem not entirely novel, and make us feel, in turn, never too alone.

## Data Availability

No datasets were generated or analysed during the current study.

## Footnotes

1 Interestingly, Projit Bihari Mukharji uses Chakabarty’s reading of Tarini to argue that the astrologer’s “imaginations of the body and disease” was an example of the “radical difference” between the “subaltern and bourgeois imaginations of the body and disease had operated in colonial India” (Mukharji 2017).

2 *Bengalee*, 30th April 1898, p. 209, cited from Harrison (1994, pp. 218–219).

3 Cited from Bose (2023). “Languages of Disease: Experiences of Plague in Late Colonial Calcutta 1890–1920” (Master's thesis, University of Oxford), p. 44. It is important to note that in this period, while some sources, like Cook’s, argued that the pandemic was a sacrifice for the goddess Kali, it was also argued by some others that both the pandemic and the vaccine were forms of the Goddess. While Kāli-as-pandemic was explained through Hindu mythological narratives such as the *Purāṇas*—where the goddess was said to descend upon the sinful world as a pandemic herself—Kāli-as-vaccine was explained through the mythological story of the slaying of *Raktabīja*, a demon who was killed by the goddess Kāli, when Kāli drank its blood and became the demon herself, thus killing him. This ambivalence was possible due to the paradox of Kāli worship itself, which saw the goddess as locus of both despair and hope, as chaos, and “deliverance from that very chaos” (Bose 2023).

4 Cited from Arnold (1993).

5 The word is “*śantilābha*”, which means the attainment of salvation and peace in a particularly Hindu idiom.

6 Carlo Caduff (2015) has written about how the language of a potential pandemics and consequent preparation is often couched in a language of anticipation, and within the landscape of a “perhaps”; this “modality of a maybe” allows governments, scientists and policymakers to institute a state of perpetual preparation for an impending apocalypse, as even if the impending catastrophe never arrives, one can always be in anticipation of the next one, and the state of preparation remains, paradoxically, constant. The case that Caduff explores is the way in which warnings about an influenza pandemic were channelled through such a “perhaps” framing. In this framing, the “perhaps” is a generative yet anxious category, prodding action through worry. Although I do not compare the two, it is interesting to note here how our astrologer somewhat flips this framing of anticipating disaster. In contrast to Caduff’s formulation (which argues that greater work and money was put into “preparedness” in the name of an epidemic disaster which was expected to arrive), in our astrologer’s case, work in vaccines *during* a pandemic was proleptic and anticipatory, but in a *positive* way. Thus, here, it was a positive “perhaps”—undertaking vaccine research during a pandemic mean that the community would, perhaps, surely recover someday soon.

7 Some of the other causes for resistance, according to this survey, included fears that the vaccine could cause impotence, have serious side effects, reduce fertility, or even cause death.

## References

[CR30] Mukharji, Projit Bihari. 2017. *Doctoring Traditions: Ayurveda, Small Technologies, and Braided Sciences*. Chicago: The University of Chicago Press.

[CR31] Navin, Mark. 2015. *Values and Vaccine Refusal: Hard Questions in Ethics, Epistemology, and Health Care*. New York: Routledge.

[CR32] Offit, Paul A., et al. 2002. Addressing Parents’ Concerns: Do Multiple Vaccines Overwhelm Or Weaken the Infant’s Immune System? *Pediatrics* 109 (1): 124–129. 10.1542/peds.109.1.124. 11773551

[CR33] Paul, Sheuli, et al. 2024. Confronting Vaccine Hesitancy in India: A Vital Call for Unified Action. *Cureus* 16 (11): e73459. 10.7759/cureus.73459. 39664158 PMC11633846

[CR500] Rodriguez, Nathan J. 2016. Vaccine-Hesitant Justifications: Too Many, Too Soon, Narrative Persuasion, and the Conflation of Expertise. *Global Qualitative Nursing Research* 3: 2333393616663304. 10.1177/2333393616663304. PMC5415268 28508015

[CR34] Rosenberg, Charles. 1999. Meanings, Policies, and Medicine: On the Bioethical Enterprise and History. *Daedalus* 128:27–46. 11624151

[CR35] Stevens, Rosemary, Charles E. Rosenberg, and Lawton R. Burns. 2006. *History and Health Policy in the United States: Putting the Past Back in*, 1st ed. New Brunswick: Rutgers University Press.

[CR36] Tashi, Kelzang T. 2022. Buddhist Rituals and COVID Vaccination in Bhutan. *LSE Blog*, The London School of Economics and Political Science, October 10, 2022. https://blogs.lse.ac.uk/southasia/2022/10/10/buddhist-rituals-and-covid-vaccination-in-bhutan/.

[CR37] Umakanthan, Srikanth, et al. 2021. COVID-19 Vaccine Hesitancy and Resistance in India Explored Through a Population-Based Longitudinal Survey. *Vaccines* 9 (10): 1064. 10.3390/vaccines9101064. 34696172 PMC8537475

[CR38] Wróblewski, Michał, and Andrzej Meler. 2024. Political Polarization May Affect Attitudes Towards Vaccination. An Analysis Based on the European Social Survey Data from 23 Countries. *European Journal of Public Health* 34 (2): 375–379. 10.1093/eurpub/ckae002. 38276887 PMC10990513

[CR39] Celebrities May Have Helped Shape Anti-vaccine Opinions During COVID-19 Pandemic, Study Finds. CNN, February 24, 2023. https://edition.cnn.com/2023/02/24/health/covid-vaccine-celebrity-influence-study/index.html.

[CR40] Gavi. 2025. *How Celebrity Endorsements Can Shape Public Health*. Gavi, the Vaccine Alliance. https://www.gavi.org/vaccineswork/how-celebrity-endorsements-can-shape-public-health.

[CR41] Pandemic Sciences Institute, University of Oxford. 2024.* Science Museum’s Injecting Hope Exhibition Enters Final Week*. Pandemic Sciences Institute, University of Oxford. https://www.psi.ox.ac.uk/news-and-opinion/science-museum2019s-injecting-hope-exhibition-enters-final-week.

[CR42] The Ex-Pfizer Scientist Who Became an Anti-vax Hero. *Reuters*, March 18, 2021. https://www.reuters.com/investigates/special-report/health-coronavirus-vaccines-skeptic/.

[CR1] Adam, Maya, et al. 2022. Hope as a Predictor for COVID-19 Vaccine Uptake in the US: a Cross-Sectional Survey of 11,955 Adults. *Human Vaccines and Immunotherapeutics* 18 (5): 2072138. 10.1080/21645515.2022.2072138. 35659447 PMC9359385

[CR2] Anderson, Katharine. 2005. *Predicting the Weather: Victorians and the Science of Meteorology*. Chicago: University of Chicago Press.

[CR5] Arnold, David. 1988. Touching the Dead: Perspectives on the Indian Plague 1896–1900. In *Selected Subaltern Studies*, ed. Ranajit Guha and Gayatri Chakravorty Spivak, 391–427. OUP: Delhi.

[CR3] Arnold, David. 1993. *Colonizing the Body: State Medicine and Epidemic Disease in Nineteenth-Century India*. Berkeley: University of California Press.

[CR4] Arnold, David. 2016. *Toxic Histories: Poison and Pollution in Modern India*. Cambridge: Cambridge University Press.

[CR7] Bhattacharya, Sanjoy, Mark Harrison, and Michael Worboys. 2005. *Fractured States: Smallpox, Public Health and Vaccination Policy in British India, 1800–1947*. Hyderabad: Orient Longman.

[CR8] Bhattacharya, S., A. Medcalf, and A. Ahmed. 2020. Humanities, Criticality and Transparency: Global Health Histories and the Foundations of Inter-sectoral Partnerships for the Democratisation of Knowledge. *Humanities and Social Sciences Communications* 7:6. 10.1057/s41599-020-0491-7. 33241231 PMC7116415

[CR9] Bose, U. 2023. Languages of Disease: Experiences of Plague in Late Colonial Calcutta 1890–1920. Master's Thesis, University of Oxford.

[CR10] Caduff, Carlo. 2015. *The Pandemic Perhaps: Dramatic Events in a Public Culture of Danger*, 1st ed. Berkeley: University of California Press.

[CR13] Chakrabarti, Pratik. 2012. *Bacteriology in British India: Laboratory Medicine and the Tropics*. Rochester: University of Rochester Press.

[CR11] Chakrabarty, Dipesh. 1981. Communal Riots and Labour: Bengal’s Jute Mill-Hands in the 1890s. *Past and Present* 91: 140–169. http://www.jstor.org/stable/650521.

[CR12] Chakrabarty, Dipesh. 2012. Community, State and the Body: Epidemics and Popular Culture in Colonial India. In Hardiman, David, and Projit Bihari Mukharji (eds). 2012. Medical Marginality in South Asia: Situating Subaltern Therapeutics. Abingdon: Routledge, 36–59.

[CR14] Chatterjee, Partha. Two Poets and Death: On Civil and Political Society in the Non-Christian World. In *Questions of Modernity*, edited by Timothy Mitchell, New Edition (Vol. 11), 35–48. University of Minnesota Press, 2000.

[CR15] Conis, Elena. 2014. *Vaccine Nation: America’s Changing Relationship with Immunization*. Chicago: University of Chicago Press.

[CR16] Cunningham, Andrew, and Ole Peter Grell. 2000. *The Four Horsemen of the Apocalypse: Religion, War, Famine and Death in Reformation Europe*. Cambridge: Cambridge University Press.

[CR17] Das, Kumar, and Bijeta Mohanty. 2021. *The Growing Concerns Regarding Vaccine Hesitancy in India*. IGC. https://www.theigc.org/blogs/growing-concerns-around-vaccine-hesitancy-india.

[CR18] Durbach, Nadja. 2005. *Bodily Matters: The Anti-vaccination Movement in England, 1853–1907*. Durham: Duke University Press.

[CR19] Flanigan, Jessica. 2014. A Defense of Compulsory Vaccination. *HEC Forum: An Interdisciplinary Journal on Hospitals’ Ethical and Legal Issues* 26 (1): 5–25. 10.1007/s10730-013-9221-5. 23942781

[CR20] Frampton, Sally, Kingsley Orievulu, Philippa C. Matthews, Alberto Giubilini, Joshua Hordern, Lizzie Burns, Sean Elias, et al. 2024. Pandemic Preparedness: Why Humanities and Social Sciences Matter. *Frontiers in Public Health* 12:1394569. 39220463 10.3389/fpubh.2024.1394569 PMC11361931

[CR21] Gantenbein, Leo Urs. 2017. Poison and Its Dose: Paracelsus on Toxicology. In *Toxicology in the Middle Ages and Renaissance*, ed. Philip Wexler. London: Elsevier/Academic.

[CR22] Gellin, B. G., et al. 2000. Do Parents Understand Immunizations? A National Telephone Survey. *Pediatrics* 106 (5): 1097–1102. 10.1542/peds.106.5.1097. 11061781

[CR24] Harrison, Mark. 1994. *Public Health in British India: Anglo-Indian Preventive Medicine 1859–1914*. Cambridge: Cambridge University Press.

[CR25] Hausman, Bernice L. 2019. *Anti/Vax: Reframing the Vaccination Controversy*. Ithaca: ILR Press, an imprint of Cornell University Press.

[CR26] Hobson-West, P. 2017. ‘Trusting blindly can be the biggest risk of all’: Organised Resistance to Childhood Vaccination in the UK. *Sociology of Health and Illness* 29:198–215. 10.1111/j.1467-9566.2007.00544.x. 17381813

[CR27] Lachenal, Guillame, and Gaëtan Thomas. 2020. COVID-19: When History Has No Lessons. In *History Workshop*, March 30, 2020.

[CR28] Liu, Yan. 2021. *Healing with Poisons: Potent Medicines in Medieval China*. Seattle: University of Washington Press.

[CR29] Mukharji, Projit Bihari. 2009. *Nationalizing the Body: The Medical Market, Print, and Daktari Medicine*. London: Anthem Press.

[CR43] Dhur, Gobin Chunder. 1898. *The Plague: Being a Reprint of Letters Published in the Indian Mirror for Allaying Popular Alarm and Conciliating the People to the Action of the Authorities*. Calcutta: Gobin Chunder Dhur.

[CR45] Jyotishi, Tarini Prasad. 1899. *Plague-Sanhita Ba Aryaswasthabidhan/The Plague-Sanhita or The Aryan Hygiene*. Calcutta: Keshablal Ray. https://indianculture.gov.in/reports-proceedings/palaega-samhaitaa-baa-araya-sabaasathabaidhaana.

[CR46] Jyotishi, Tarini Prasad, and Delhi Coronation Darbar. 1911. *Ascension of King George V to the Throne (a Melody)/Pancham Georger Singhasanarohan*, 1911. Calcutta: Surendra Nath Guha.

[CR44] Leigh Gardner, F 1902. *A Catalogue Raisonne of the Works on the Occult Sciences: Astrological Books*, vol. II. London: Cambridge University Press.

[CR47] A Bengali Zadkiel. *The Times of India (1861–2010)*, April 15, 1892. https://www.proquest.com/historical-newspapers/bengali-zadkiei/docview/234335677/se-2.

[CR48] An Astrologer's. *The Times of India (1861–2010)*, March 1, 1897. https://www.proquest.com/historical-newspapers/astrologers/docview/234148032/se-2

[CR49] An Indian Zadkiel: Some of the Things He Prophesied Have Really Come to Pass. Kansas City Journal (Kansas City, Mo.), 23 July 1899. In *Chronicling America: Historic American Newspapers*. Library of Congress. https://chroniclingamerica.loc.gov/lccn/sn86063615/1899-07-23/ed-1/seq-18/.

[CR50] An Undiscount Aged Prophet. *The Times of India (1861–2010)*, April 19, 1895. https://www.proquest.com/historical-newspapers/undiscount-aged-prophet/docview/234147266/se-2.

[CR51] Books of the Week, New-York Tribune (New York [NY]), 25 July 1903. *Chronicling America: Historic American Newspapers*. Library of Congress. https://chroniclingamerica.loc.gov/lccn/sn83030214/1903-07-25/ed-1/seq-10/.

[CR52] Dr. Nield Cook and Plague Precautions in Calcutta: Haffkine's Prophylactic Money Must Be Freely Expended Stamping Out the Disease by Repressive Measures the Infection of Rats Family Hospitals. *The Times of India (1861–2010)*, April 29, 1898. https://www.proquest.com/historical-newspapers/dr-nield-cook-plague-precautions-calcutta/docview/234114508/se-2.

[CR53] Local Echoes. *The Derby Mercury*, June 28, 1899. The British Newspaper Archive.

[CR54] The New Viceroy: A Prediction. *The Times of India (1861–2010)*, January 7, 1899. https://www.proquest.com/historical-newspapers/new-viceroy/docview/311073994/se-2.

[CR55] The Bengali Zadkiel. *The Times of India (1861–2010)*, February 1, 1898. https://www.proquest.com/historical-newspapers/bengali-zadkiel/docview/231223688/se-2.

[CR56] "The Bengali Zadkiel Interviewed: His Prediction Correct. *The Times of India (1861–2010)*, June 22, 1897. https://www.proquest.com/historical-newspapers/bengali-zadkiel-interviewed/docview/234126153/se-2.

[CR57] The Prophet of Bengal. *The Times of India (1861–2010)*, April 27, 1893. https://www.proquest.com/historical-newspapers/prophet-bengal/docview/234187370/se-2.

